# Outcomes of Endoscopic Resection of Circumferential Colorectal Laterally Spreading Lesions: A Western Experience

**DOI:** 10.3390/diagnostics15192534

**Published:** 2025-10-08

**Authors:** Gianluca Andrisani, Mattia Brigida, Giulio Antonelli, Cesare Hassan, Chiara Taffon, Andrea D’Amico, Virginia Gregorio, Giovanni Parente, Michele Cicala, Antonio Facciorusso, Francesco Maria Di Matteo

**Affiliations:** 1 Digestive Endoscopy Unit, University Campus Bio-Medico, 00128 Rome, Italy; gianluca.andrisani@gmail.com (G.A.); mattiabrigida@hotmail.it (M.B.); andrea.damico@unicampus.it (A.D.); virginia.gregorio01@universitadipavia.it (V.G.); f.dimatteo@policlinicocampus.it (F.M.D.M.); 2 Gastroenterology Unit, Department of Experimental Medicine, Università del Salento, Piazza Filippo Muratore 1, 73100 Lecce, Italy; 3 Gastroenterology and Digestive Endoscopy Unit, Ospedale dei Castelli (N.O.C.), 00040 Rome, Italy; giulio.antonelli@gmail.com; 4 Endoscopy Unit, Humanitas Clinical and Research Center IRCCS, 20089 Rozzano, Italy; cesare.hassan@humanitas.eu; 5 Pathology Unit, Fondazione Policlinico Universitario Campus Bio-Medico, 00128 Rome, Italy; c.taffon@policlinicocampus.it; 6 Unit of Gastroenterology, Università Campus Bio-Medico di Roma, 00128 Rome, Italy; m.cicala@policlinicocampus.it

**Keywords:** endoscopic submucosal dissection, circumferential laterally spreading lesions, near-circumferential laterally spreading lesions, post-ESD stricture, colorectal ESD

## Abstract

**Background**: Circumferential or near-circumferential colorectal lesions are challenging to remove endoscopically; therefore, they are often surgically managed. There are limited data on the outcomes of endoscopic submucosal dissection (ESD) for these lesions, usually from Eastern settings, where ESD is more well established. **Objective**: The objective of the study was to retrospectively analyze the outcomes of circumferential colorectal ESD in a Western center. **Methods**: Consecutive patients referred for endoscopic resection of colorectal lesions between January 2015 and April 2025 were included if they had undergone ESD for colorectal laterally spreading tumors with ≥90% involvement of the luminal circumference. **Results**: Overall, 53 patients were enrolled (26 females, 49.1%; 70.6 ± 9.3 years). Mean lesion size was 91.8 ± 25.3 mm. The most frequent lesion location was the rectum (*n* = 36, 67.9%). Thirty-three lesions (62.3%) were circumferential, and twenty (37.7%) were near-circumferential. Median procedural time was 160.0 min (IQR 112.0–200.0 min). Histological analysis revealed high-grade dysplasia in 25/53 cases (47.2%) and adenocarcinoma in 28 patients (52.8%). Resection was en bloc in 51 cases (96.2%) and R0 in all cases (100%). Curative resection was achieved in 21 out of 28 adenocarcinoma patients (75%). Adverse events were intra-procedural major bleeding (*n* = 19, 18.9%), post-procedural bleeding (*n* = 2, 3.8%), delayed bleeding (*n* = 1, 1.9%), and intra-procedural perforation (*n* = 3, 5.7%). Post-ESD stricture was observed in 18.9% of cases (10/53); three of these (30%) were asymptomatic. All were successfully managed endoscopically. Patients who developed strictures had a longer median procedural time (206 min vs. 145 min, *p* = 0.0061) and a larger mean lesion size (110 mm vs. 90 mm, *p* = 0.035). **Conclusions**: ESD for colorectal circumferential and near-circumferential lesions was safe and effective in a Western expert center, supporting the use of this technique in this subset of lesions. Strictures are a common consequence that can be effectively managed endoscopically.

## 1. Introduction

Endoscopic submucosal dissection (ESD) is an advanced endoscopic technique that enables en bloc resection of large colorectal lesions [1,2]. This approach allows for detailed histopathological assessment and has demonstrated potential advantages over surgical resection, including lower morbidity, reduced mortality, and shorter recovery times. Compared with endoscopic mucosal resection (EMR), ESD offers the benefit of en bloc resection, which provides more complete pathological evaluation and reduces the risk of local recurrence [3]. Recent multicenter trials and large cohort studies have reported procedural success rates exceeding 85–90%, with en bloc and R0 resection rates superior to conventional endoscopic mucosal resection, even for large laterally spreading tumors [4,5].

Beyond oncologic efficacy, ESD has been associated with reduced need for surgery, lower overall treatment costs, and high levels of patient acceptance compared with surgical resection [6].

These advantages underscore the growing role of colorectal ESD as a minimally invasive, organ-preserving therapy, particularly in high-volume centers with appropriate expertise [4]. Despite these advantages, many patients with large colorectal adenomas that do not show evidence of invasive cancer continue to undergo radical surgery [3].

This is particularly true for technically complex lesions, such as circumferential or near-circumferential adenomas, which may represent a significant portion of surgically managed cases. Establishing the safety and efficacy of ESD for these lesions is, therefore, essential. However, even in high-volume tertiary centers, the overall incidence of such lesions is low, and published data from Japan regarding ESD for circumferential or near-circumferential lesions remain scarce [7,8,9].

Although the use of ESD is increasing in expert centers across Western countries, there are limited data on the outcomes of ESD for these specific lesion types in Western populations. In particular, post-procedural stricture is one of the late adverse events occurring after an ESD involving more than 75% of lumen circumference [7,8,9], but to date, there is still a knowledge gap on the frequency of this event, especially for sub-circumferential (90 to 99% of lumen circumference) or circumferential (100% of the lumen involved) dissected lesions.

The aim of this study was to analyze the outcomes of circumferential colorectal ESD performed in a Western expert center, focusing on post-ESD stricture occurrence and management.

## 2. Materials and Methods

### 2.1. Study Design

This was a retrospective, single-center study conducted in the Digestive Endoscopy Unit of Fondazione Campus Bio-Medico in Rome, Italy. The study protocol conformed to the ethical guidelines of the Declaration of Helsinki (1975) and was approved by the local institutional review board (IRB No. UCBM C-ESD, 7 July 2025). Consecutive patients referred for endoscopic resection of colorectal lesions between January 2015 and April 2025 were assessed for inclusion and retrospectively enrolled if they met the study criteria. Written informed consent had been obtained from all patients prior to the procedures. Methodology of the results reporting complies with the STROBE guidelines.

### 2.2. Inclusion Criteria and Data Collection

Patients were included if they had undergone ESD for colorectal laterally spreading tumors (LSTs) with ≥90% involvement of the luminal circumference, regardless of lesion morphology or histology.

Data were collected on patient demographics, lesion characteristics, procedural details, histopathological outcomes, adverse events, recurrence, and further treatment. The extent of mucosal defect was estimated as a percentage of the colorectal circumference and categorized as <75%, 75–89%, 90–99% (near-circumferential), and 100% (circumferential). Concerning colorectal circumference involvement, the inclusion criterion was a lesion extent of >90% of the lumen circumference.

### 2.3. Definitions

In order to evaluate patient health status and general conditions, Charlson’s comorbidity index (i.e., a scoring system developed to predict 10-year mortality based on the presence and severity of various comorbid conditions, where each condition is assigned a weighted score from 1 to 6) was used [10]. The total score correlates with mortality risk. ASA scores (i.e., a system developed by the American Society of Anesthesiologists to assess and communicate a patient’s preoperative health status, where ASA I: normal healthy patient, ASA II: patient with mild systemic disease, ASA III: patient with severe systemic disease, ASA IV: patient with severe systemic disease that is a constant threat to life, ASA V: moribund patient not expected to survive without operation, and ASA VI: brain-dead patient whose organs are being removed for donor purposes) [11] were used.

Lesions were classified morphologically using the Paris classification [12] and further stratified by LST subtype (granular homogeneous, granular mixed, non-granular flat-elevated, or pseudo-depressed). Lesions were classified according to the Japan Narrow Band Imaging Expert Team (JNET) classification [13] through narrow-band imaging (NBI) or Blu Light Imaging (BLI) technology. Lesions were suspected of deep submucosal invasion based on endoscopic appearance. If the lesion was classified as JNET 2B (i.e., variable vessels’ caliber, with irregular distribution; irregular surface pattern) [13] or JNET 3 (i.e., avascular areas and amorphous surface areas) [13] or had a Kudo type V_N pit pattern (i.e., with unstructured glandular patterns) [14], it was excluded from ESD and referred for surgery.

Stricture was defined as any post-procedural luminal narrowing impeding passage of a standard colonoscope (11.8–12.9 mm outer diameter), irrespective of clinical symptoms.

Endoscopically, the degree of submucosal fibrosis was categorized based on the observation at the time of the submucosal injection [15] as follows: F0 (no fibrosis): submucosal layer appears as a transparent, normal submucosa without any fibrotic changes; F1 (mild fibrosis): submucosal layer shows a white, web-like appearance of fibrosis dispersed within an otherwise transparent layer, and dissection is feasible but requires more careful technique; and F2 (severe fibrosis): submucosal layer appears as a dense, white, muscle-like structure without transparency, and this indicates marked fibrosis, often making dissection very difficult and associated with higher risk of incomplete resection or complications.

### 2.4. Endoscopic Procedures

All procedures were performed by experienced endoscopists (i.e., who have performed >100 colorectal ESD or >25 ESD/year) [16,17]. Lesions were evaluated using virtual chromoendoscopy and magnification colonoscopy (EG-760 and EC-760PL, Fujifilm, Tokyo, Japan). All lesions without evidence of invasive cancer beyond T1 were considered for ESD. Procedures were performed under deep sedation or general anesthesia (with orotracheal intubation), and all patients were hospitalized for a minimum of two nights post-procedure.

ESD was performed using a therapeutic endoscope (EG-760CT or EC-760PL, Fujifilm) equipped with a tapered hood, electrosurgical knives (FlushKnife, GoldKnife, or HybridKnife), and monopolar hemostatic forceps (Ensure, Micro-Tech (Nanjing) Co., Ltd., Nanjing, China). A high-frequency electrosurgical unit (VIO 300D or VIO 3, ERBE Elektromedizin GmbH, Tübingen, Germany) was used. Submucosal injection solutions included saline, adrenaline, and indigo carmine. The technique used as first choice was submucosal tunneling (i.e., a technique in which a submucosal tunnel is created beneath the lesion by making two mucosal entry points, the proximal and distal or oral and anal sides, and then dissecting the submucosal layer between them) [18,19] or the pocket creation method (i.e., a variation of ESD where a submucosal pocket is created under the lesion by limiting the initial mucosal incision to a small entry and then expanding the submucosal space beneath the lesion before extending the mucosal incision) [20]. These techniques, when applied for resection of lesions involving 90–100% of lumen circumference in our cohort, are defined as circumferential ESD for the purposes of this study. The other two variants of ESD sometimes used in our cohort are underwater ESD (when the lumen is completely filled with saline during ESD in order to have better dissection plans and a better view of the vascularization) [21,22] and intermuscular dissection (i.e., for rectal lesions, complete submucosal dissection by gaining access to the muscular layer) [23,24]. Surveillance colonoscopies were scheduled at 3–6 months and 12 months post-resection.

If a stricture developed during the follow-up period, it could either undergo endoscopic balloon dilation or Savary dilation with a maximum caliber of 20 mm, with the possibility of repeating the dilation treatment as needed.

### 2.5. Histopathology Assessment

**Figure 1:**
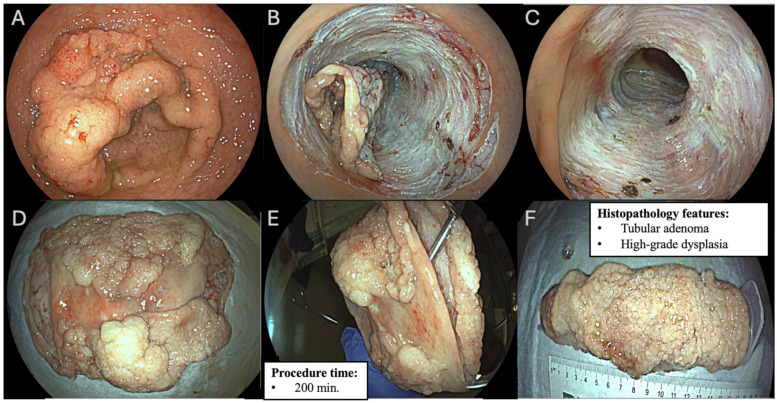
Laterally spreading tumor and granular mixed subtype (LST-GM) involving 100% of rectal lumen circumference: (**A**) lesion before circumferential endoscopic submucosal dissection (ESD); (**B**) lesion surface subtotal detachment from deeper submucosal plane and exposed submucosal vessels before preventive coagrasper coagulation; (**C**) exposed submucosal layer following circumferential ESD, where the muscularis propria is preserved and no signs of deep mural injury are evident; (**D**,**E**) circumferential specimen; (**F**) long axis size measurement of circumferential specimen (160 mm).

**Figure 2:**
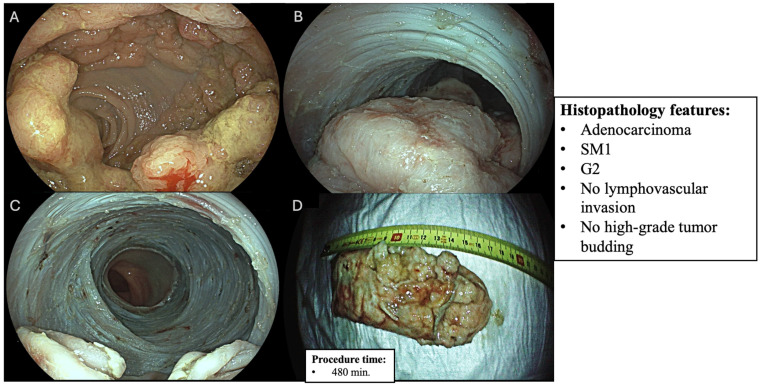
Laterally spreading tumor and granular mixed subtype (LST-GM) involving 100% of colonic lumen circumference: (**A**) lesion before circumferential endoscopic submucosal dissection (ESD); (**B**) endoscopic view after approximately 60% of the lesion has been dissected, where the partial submucosal exposure is displayed on the gravity-dependent side with the dissected area appearing clean and well-demarcated; (**C**) submucosal bed after ESD at the moment of lesion retrieval; (**D**) long axis size of the resected specimen (170 mm).

Specimens (whose examples are shown in Figure 1 and Figure 2) were fixed on cork, stretched with the mucosal side exposed, and immersed in 4% buffered formalin. Histopathological analysis was performed by a dedicated gastrointestinal pathologist according to the Vienna classification [25,26]. The specimens (biopsies or resections) are interpreted by a pathologist specialized in gastrointestinal tract pathology (higher expertise, experience with GI epithelial neoplasia) rather than a general pathologist; the pathologist classifies the lesions using the Vienna categories from the criteria (i.e., negative/indefinite/low-grade/high-grade/invasive) based on morphology (architecture, cytologic atypia, invasion) using standardized definitions.

The following features were recorded: lesion size, depth of invasion, resection margins (R0, R1, Rx), presence of lymphovascular invasion, and tumor budding.

Submucosal invasion ≥ 1000 µm (SM2) was considered deep and non-curative. Curative resection was defined as en bloc R0 resection, submucosal invasion < 1000 µm, absence of lymphovascular invasion, tumor budding grade 1, and well-to-moderately differentiated histology (G1–G2).

### 2.6. Study Outcomes

The primary outcome was the definition of the en bloc and R0 resection rates.

Secondary outcomes were the following:

- Incidence of post-ESD stricture in circumferential and near-circumferential colorectal lesions.
- Procedure time and dissection speed (defined as specimen area divided by procedure duration).
- Incidence of adverse events (intra- and post-procedural bleeding, perforation, PECS).
- Identification of clinical and procedural predictors of stricture development.

Specimen area was calculated using the formula for the area of an ellipse as follows:

Area = (short axis/2) × (long axis/2) × π.

### 2.7. Definitions of Adverse Events and Follow-Up

Procedure-related adverse events (AEs) were intra-procedural bleeding, late bleeding, perforation, and post-ESD electrocoagulation syndrome (PECS). Severity of adverse events was classified according to the American Society of Gastrointestinal Endoscopy (ASGE) lexicon [27].

- Intra-procedural bleeding was defined as any bleeding that occurs during the ESD procedure itself [27,28].
- Late bleeding was defined as a reduction in total hemoglobin value of more than 2 g/dL from preoperative levels or as the finding of massive post-procedure melena in the absence of other apparent sources of bleeding [27,28].
- Perforation was defined as a complication if it occurred during the procedure or was found later by an abdominal RX or CT scan [27,28].
- PECS was diagnosed based on [27,28] the following:
  1. The presence of abdominal distension at the site of endoscopic resection.
  2. The presence of fever (>37.5 °C) or increased indices of inflammation (C-reactive protein > 0.5 mg/dL or leukocytosis > 10,000 cells/μL), without evidence of established perforation arising > 6 h after the endoscopic procedure.

### 2.8. Statistical Analysis

Continuous variables were described using mean ± standard deviation or median with interquartile range, and categorical variables as counts and percentages. Comparisons between patients with and without post-ESD stricture were performed using Fisher’s exact test for categorical variables and the Mann–Whitney U test for continuous variables.

A multivariate logistic regression model was constructed to identify independent predictors of stricture formation, including variables with *p* < 0.10 at univariate analysis. Model discrimination was assessed with the area under the ROC curve, and odds ratios with 95% confidence intervals were reported.

A linear regression model was used to evaluate predictors of procedural time. Statistical significance was set at *p* < 0.05. Analyses were performed using MedCalc (version 23.2.0, Ostend, Belgium) and R software (version 4.5.1, Vienna, Austria).

## 3. Results

### 3.1. Patient Characteristics

A total of 72 patients potentially eligible for the study were identified. After the exclusion of 19 patients with lesions < 90% of lumen circumference, 53 patients who underwent colorectal ESD for lesions involving ≥90% of the luminal circumference were finally included in the analysis. The mean age was 70.6 ± 9.3 years, and 26 were females (49.1%). Full demographic and clinical data are summarized in Table 1.

Overall health status and risk before anesthesia and ESD were assessed via the ASA classification. A total of 28 patients scored ASA I (52.8%) and 25 patients scored ASA III (47.2%), while no patient scored ASA II, IV, V, or VI. Concerning antithrombotic therapy, six patients (11.3%) were under anticoagulant treatment, fourteen patients (26.4%) were under antiplatelet treatment, and three (5.6%) were under both treatments.

### 3.2. Procedural and Lesion Characteristics

The mean lesion size was 91.8 + 25.3 mm. Thirty-three lesions (62.3%) were classified as circumferential, and twenty (37.7%) were near-circumferential. En bloc resection was achieved in 96.2% of cases (51/53). R0 resection was achieved in 53 patients (100%). Median procedural time was 160.0 min (IQR 112.0–200.0 min). ESD was performed using one or more of the following techniques. Overall, the underwater technique was employed for twenty-one lesions (39.6%), the tunnel technique for forty-nine lesions (92.5%), and the intermuscular dissection for two lesions (3.8%). No traction technique was performed in this series of patients.

The most frequent lesion location was the rectum (*n* = 36, 67.9%), followed by the caecum (*n* = 6, 11.3%) and, equally, ascending and transverse colon (each *n* = 4, 7.5%). In terms of endoscopic morphology, granular-type LSTs were the most common, with 5.7% classified as granular homogeneous and 90.6% as granular mixed. Non-granular flat-elevated and pseudo-depressed morphologies were observed in 3.8% and 0% of cases, respectively. According to the Japan NBI Expert Team (JNET) classification, 17 out of 53 (32.1%) of lesions were classified as JNET 2A and 36 (67.9%) as 2B. Complete data on lesion characteristics is available in Table 1.

The majority of procedures (*n* = 49, 92.5%) were performed using the tunneling technique, while the conventional technique was used in four cases (7.5%). During ESD, when fibrosis was observed (*n* = 16), it was reported as F1 in fourteen cases (26.4%) and F2 in two cases (3.8%).

Histological analysis (Table 2) revealed a diagnosis of HGD in 25 out of 53 (47.2%) of cases and adenocarcinoma in 28 patients (52.8%). Among adenocarcinomas, twenty-one (75%) were low risk with no HR features, and seven (25%) were high risk. Among risk factors, in five cases, deep submucosal invasion plus high-grade tumor budding was observed (two of these also had lympho-vascular invasion). In the other two cases, even though superficial submucosal invasion was registered, high-grade tumor budding was present.

Resection was considered en bloc in 51 cases (96.2%) and R0 in all cases (100%). Curative resection was achieved in 21/28 patients (75%) based on standard histological criteria, including absence of deep submucosal invasion, lymphovascular invasion, and tumor budding.

Recurrence was observed for six lesions (11.3%) at the moment of enrollment; in particular, recurrence after previous endoscopic piecemeal mucosal resection (EPMR) and trans-anal endoscopic microsurgery (TEM) was observed, respectively, in three (5.7%) and one (1.9%) patients. Instead, two recurrences were seen over time since the enrollment, during follow-up after circumferential ESD. These were treated with endoscopic dissection, with no need for further treatment. Data of these two patients are summarized in Table 3.

A subgroup univariate analysis was conducted in order to evaluate differences between lesions involving 90% vs. 100% of lumen circumference (summarized in Table 4). No statistically significant differences were detected between the two groups concerning demographic, endoscopic, and histologic outcomes.

### 3.3. Stricture Formation and Treatment

A post-ESD stricture development was observed in 18.9% of cases (10/53). Out of these, eight patients (80%) developed rectal stricture, one patient (10%) developed anal stricture, and one patient (10%) developed both rectal and anal stricture. No colonic strictures were observed. Among the 10 patients who developed a stricture, three (30%) were asymptomatic, while for patients developing symptoms (70%), the median time to symptoms was 90 days (IQR 45–109).

Patients who developed strictures had a significantly longer median procedural time compared to those who did not (206 min vs. 145 min, *p* = 0.0061), as well as a larger mean lesion size (110 mm vs. 90 mm, *p* = 0.035)—see Table 5.

On univariate analysis, larger lesion size (*p* = 0.0396) and longer procedural time (*p* = 0.0061) were associated with an increased risk of stricture formation. Other variables, including prior colorectal surgery, presence of submucosal fibrosis, histologic diagnosis (adenoma vs. adenocarcinoma), and resection technique, were not significantly associated with stricture development.

A multivariate logistic regression model including lesion size, procedural time, and degree of circumferential involvement did not identify any independent predictors of stricture formation (Table 6).

All patients with stricture underwent endoscopic treatment: 50% (*n* = 5) received repeated endoscopic balloon dilation, 40% (*n* = 4) received repeated Savary dilation, and 10% (*n* = 1) received both repeated endoscopic dilations and LAMS placement (Axios). The mean number of endoscopic balloon dilations performed was 2.3 ± 1.1, whilst the mean number of Savary dilations performed was 2.8 ± 1.

### 3.4. Adverse Events

Overall, adverse events in circumferential ESD occurred in 16 cases (Table 7). Intra-procedural bleeding was observed in 10 patients (18.9%), and they were classified as mild according to ASGE severity grading. In all of these cases, hemostasis was reached with coagrasper. Mild intra-procedural perforation occurred in three cases (5.7%), and in all of these cases, it was successfully managed with TTS/OTSC clips.

Post-procedural bleeding occurred in two cases (3.8%), and they were moderate according to the ASGE severity grading system. One spontaneously resolved without needing treatment, and the other was treated with coagrasper. Delayed bleeding was observed in one patient (1.9%); it was moderate according to ASGE severity grading, and it was treated with coagrasper.

No post-procedural or delayed perforation was observed, and no other hemostatic devices were needed [29].

## 4. Discussion

This study presents the largest Western series to date focused exclusively on endoscopic submucosal dissection (ESD) of colorectal laterally spreading tumors (LSTs) with circumferential or near-circumferential extension. Our findings confirm that ESD is technically feasible in this challenging subgroup of lesions, with acceptable rates of adverse events and promising histological outcomes when performed in expert centers. Importantly, we provide a detailed analysis of stricture formation—a feared complication in this setting—and propose early insights into potential procedural risk factors.

Strictures occurred in approximately one-fifth of cases (18.9%), a rate consistent with that reported in smaller Eastern series of similar lesions [8,9]. Notably, a substantial proportion of these strictures (30%) were asymptomatic and detected incidentally during scheduled follow-up endoscopy. This finding raises the question of whether routine endoscopic surveillance, rather than preemptive dilation strategies, may suffice in the absence of symptoms. In symptomatic cases, endoscopic dilation was effective in restoring luminal patency, although a minority of patients required multiple sessions.

Our analysis identified lesion size and procedural time as factors associated with stricture formation at univariate analysis. These variables likely reflect technical complexity, submucosal fibrosis, and prolonged cautery exposure—all contributing to deeper mural injury and subsequent fibrosis. However, no independent predictors emerged in multivariate analysis. Despite the high discriminative performance of our regression model (AUC 0.91), it should be considered exploratory, as the lack of statistical significance for individual predictors reflects the study’s limited power and the collinearity between included variables (e.g., long axis size correlating with procedural time, which accounts for collinearity resulting in the absence of identification of a significant predictor at the multivariate analysis).

Most lesions in our series were located in the rectum and resected using the tunneling technique, which may have facilitated en bloc resection and minimized the extent of circumferential submucosal fibrosis at any given plane. With en bloc and curative resection rates comparable to non-circumferential ESD series, these results suggest that extensive colorectal lesions need not be considered a contraindication to endoscopic treatment in high-volume centers. The predominance of rectal LSTs also aligns with referral patterns and lesion prevalence in tertiary practice but may limit generalizability to lesions in more proximal locations, where strictures may have different clinical behavior and technical constraints. In this context, the pocket creation method represents another validated approach to facilitate safe and efficient dissection of large colorectal tumors [20]. By maintaining a limited mucosal incision and creating a submucosal pocket, the pocket creation method improves traction and stability, reduces insufflation leakage, and has been associated with higher en bloc resection rates and shorter procedure times compared with conventional ESD. Although our series relied primarily on tunneling, both techniques highlight the value of strategies that preserve submucosal exposure and counter-traction in managing extensive lesions.

When comparing ESD with surgical approaches, such as laparoscopic or robot-assisted resection for large colorectal laterally spreading tumors, several differences have been reported. ESD is generally associated with longer procedural times than laparoscopic surgery, particularly for circumferential or near-circumferential lesions, reflecting the technical complexity of submucosal dissection [30]. However, this is offset by its minimally invasive profile, with lower perioperative morbidity and adverse event rates compared with colectomy, including fewer cardiopulmonary and infectious complications [4,31].

Importantly, ESD provides significant advantages in post-procedural recovery, with shorter hospital stays—often 3–5 days versus 7–10 days for laparoscopic colectomy—and rapid return to daily activities [5,31].

From an economic perspective, several analyses have demonstrated that ESD is more cost-effective than surgery, largely by avoiding operating room costs, general anesthesia, and prolonged hospitalization, despite the longer procedure duration [5,6].

Taken together, these findings indicate that while technically demanding, colorectal ESD—when feasible in high-volume centers—offers a favorable balance of safety, recovery, and cost compared with surgical resection, even in the setting of extensive or circumferential lesions.

Several limitations must be acknowledged. First, the retrospective nature of the study introduces inherent risks of selection bias and missing data. Nonetheless, all cases were drawn from a prospectively maintained ESD registry, and no eligible patients were excluded, which limits the potential for bias. Second, although the cohort represents the largest Western experience in this specific lesion type, the overall sample size remains modest, and statistical analyses—particularly multivariable modeling—should be interpreted cautiously. The associations identified are best regarded as hypothesis-generating and require validation in larger, prospective series.

Lastly, follow-up was limited to the post-procedural period necessary to detect strictures and early adverse events. Long-term oncological outcomes, including recurrence or metachronous lesion development, were not assessed in this study and warrant further investigation. Follow-up of these patients will probably be extended in order to better assess post-procedural adverse events over time.

In conclusion, ESD for colorectal circumferential and near-circumferential laterally spreading tumors is feasible and effective in expert centers, with high rates of en bloc and curative resection. Stricture formation remains a relatively frequent adverse event in this setting but is often asymptomatic and manageable with endoscopic therapy. Larger lesion size and longer procedural time may be associated with increased stricture risk, although current evidence remains exploratory. These findings also support the use of ESD as a valid organ-preserving strategy for extensive colorectal lesions and underscore the need for close endoscopic follow-up.

## Figures and Tables

**Table 1:** Demographics and clinical and endoscopic data.

Variables	n (%)
**Demographics and clinical data**	
**Number of patients enrolled**	53 (100%)
**Sex (females)**	26 (49.1%)
**Mean age (years)**	70.6 ± 9.3
**Charlson Comorbidity Index (CCI)**	
• CCI 0	2 (3.8%)
• CCI 1–2	13 (24.6%)
• CCI 3–5	37 (69.7%)
• CCI > 5	1 (1.9%)
**ASA classification**	
• ASA-I	28 (52.8%)
• ASA-II	0 (0%)
• ASA-III	25 (47.2%)
**Antithrombotic treatment**	
• Anticoagulant	6 (11.3%)
• Antiplatelet	14 (26.4%)
• Both	3 (5.6%)
**Endoscopy: lesion assessment and procedural details**	
**Lesion location**	
• Rectum	36 (67.9%)
• Rectosigmoid	3 (5.7%)
• Sigmoid	1 (1.9%)
• Descending	0 (0%)
• Transverse	4 (7.5%)
• Ascending	4 (7.5%)
• Caecum	6 (11.3%)
**Mean lesion size (mm)**	91.8 ± 25.3
**Paris classification**	
• LST-GM	48 (90.6%)
• LST-GH	3 (5.7%)
• LST-NG-F	2 (3.8%)
**JNET classification**	
• 2A	17 (32.1%)
• 2B	36 (67.9%)
**Circumferential involvement**	
• 90–99% involved	20 (37.7%)
• 100% involved	33 (62.3%)
**Fibrosis**	
• F1	14 (26.4%)
• F2	2 (3.8%)
**ESD technique**	
• Underwater	21 (39.6%)
• Tunnel	49 (92.5%)
• Intermuscular	2 (3.8%)
**General anesthesia with orotracheal intubation**	22 (41.5%)
**En bloc resection**	51 (96.2%)
**Median procedural time (min)**	160.0 (IQR 112.0–200.0)

**Table 2:** Histopathology evaluation of resected lesions.

Variables	n (%)
**Histopathology evaluation**	
**Median lesion size at histology (mm)**	90.0 (IQR 75.0–110.0)
**High-grade dysplasia**	25 (47.2%)
**Adenocarcinoma diagnosis**	28 (52.8%)
**In situ adenocarcinoma**	11 (39.3%)
**Submucosal (SM) involvement**	
• SM1	12 (42.9%)
• SM2	5 (17.8%)
**Lymphovascular invasion**	2 (7.1%)
**High-grade tumor budding**	7 (25.0%)
**Grading**	
• G1	13 (46.4%)
• G2	14 (50.0%)
• G3	1 (3.6%)
**Oncologically curative resection (out of adenocarcinoma diagnosis)**	21 (75%)

**Table 3:** Descriptive data for recurrence cases.

Variables	Recurrence Patient 1	Recurrence Patient 2
**Age**	69	61
**Sex**	M	F
**ASA**	3	1
**CCI**	4	2
**Time to recurrence**	14 months	8 months
**Lesion location**	Transverse colon	Cecum
**Lesion size**	90	65
**Morphology**	LST-GM	LST-GM
**JNET**	2B	2B
**Histology**	In situ adenocarcinoma	In situ adenocarcinoma
**Lymphovascular invasion**	None	None
**High-grade tumor budding**	None	None
**Grading**	G1–G2	G1–G2
**En bloc resection**	Yes	Yes
**R0**	Yes	Yes
**Procedure time**	210	153

**Table 4:** Subgroup univariate analysis: 90% vs. 100% lumen circumference involvement.

Variables	Group 90% Circumferential Involvement (n = 20)	Group 100% Circumferential Involvement (n = 33)	*p*-Value
**Age (years, mean ± std dev)**	69.9 ± 10.0	71 ± 9.1	0.682
**Gender (n females)**	9 (45%)	17 (51.5%)	0.859
**ASA class ≥ 3 (n)**	6 (30%)	19 (57.6%)	0.095
**Long axis size (mm)**	95.2 ± 36.8	91.9 ± 25.9	0.382
**Distal rectum involvement (n)**	6 (30%)	16 (48.5%)	0.300
**En bloc resection (n)**	1 (5%)	1 (3%)	1.000
**Procedure time (min, mean ± std dev)**	173.7 ± 93.1	156.2 ± 55.6	0.876
**Orotracheal intubation (n)**	10 (50%)	12 (36.4%)	0.490
**Adenocarcinoma (n)**	12 (60%)	16 (48.5%)	0.596
**High-grade tumor budding (n)**	3 (15%)	4 (12.1%)	1.000
**Lymphovascular invasion (n)**	2 (10%)	0 (0%)	0.138
**SM+ (n)**	7 (35%)	10 (30.3%)	0.959
**Recurrence (n)**	1 (5%)	1 (3%)	1.000
**Stricture development (n)**	5 (25%)	5 (15.2%)	0.475

**Table 5:** Univariate analysis: stricture vs. no stricture.

Variable	Stricture (n = 10)	No Stricture (n = 43)	*p*-Value
**Demographics**			
**Sex (females)**	6 (60%)	21 (48.8%)	0.7277
**Age (mean yrs ± STD)**	68.9 ± 9.9	71.0 ± 9.1	0.5332
**Endoscopic evaluation and procedural variables**			
**Long axis size (mm)**	110 (90.5–127.5)	90 (70–110)	0.035
**Circumferential involvement** • 90–100% involved • 100% involved	5 (50%) 5 (50%)	15 (34.9%) 28 (65.1%)	0.475
**Morphology (Paris)** • LST-GH • LST-GM • LST-NG-F	0 (0%) 10 (100%) 0 (0%)	3 (7.0%); 38 (88.4%) 2 (4.7%)	0.57
**JNET class** • 2A • 2B	1 (10%) 9 (90%)	16 (37.2%) 27 (62.8%)	0.1405
**Fibrosis**	4 (40%)	12 (27.9%)	0.4667
**Orotracheal intubation**	9 (90%)	13 (30.2%)	0.0008
**Procedural time (min)**	206 (170.5–236)	145 (105–182.5)	0.0061
**Intra-procedural major bleeding**	5 (50%)	5 (11.6%)	0.0138

**Table 6:** Multivariate analysis—predictive model for stricture development.

Model Variables	Odds Ratio	Lower CI	Upper CI	*p*-Value
**Const**	0.001	1.77 × 10^−8^	18.496	0.158
**Age**	1.027	0.884	1.191	0.730
**ASA class**	0.341	0.064	1.803	0.205
**Procedural time**	1.017	0.995	1.038	0.119
**Long axis size**	1.008	0.966	1.050	0.726
**Circumferential involvement**	1.722	0.194	15.274	0.625
**Distal rectum involvement**	1.588	0.223	11.294	0.644
**Orotracheal intubation**	9.749	0.683	139.115	0.093
**Intra-procedural bleeding**	3.066	0.323	29.018	0.328

Model performance metrics: AUC-ROC: 0.913953488372093; log-likelihood: −14.917967622087845; pseudo R-squared: 0.4188110251839381; LLR *p*-value: 0.005931174021010829.

**Table 7:** Intra-procedural, post-procedural, and delayed adverse events.

Variables	n (%)
**Intra-procedural, post-procedural, and delayed complications**	
**Intra-procedural major bleeding**	10 (18.9%)
**Intra-procedural perforation**	3 (5.7%)
**Post-procedural bleeding**	2 (3.8%)
**Delayed bleeding**	1 (1.9%)
**Post-procedural perforation**	0 (0%)
**Delayed perforation**	0 (0%)

## Data Availability

The raw data supporting the conclusions of this article will be made available by the authors upon request.

## Abbreviations

The following abbreviations are used in this manuscript:

ESD	Endoscopic Submucosal Dissection
EMR	Endoscopic Mucosal Resection
LST	Laterally Spreading Tumor
JNET	Japan Narrow Band Imaging Expert Team
BLI	Blu Light Imaging
ASGE	American Society of Gastrointestinal Endoscopy
SM	Submucosal
AE	Adverse Event
PECS	Post-ESD Electrocoagulation Syndrome
ASA	American Society of Anesthesiology
IQR	Interquartile Range
EPMR	Endoscopic Piecemeal Mucosal Resection
TEM	Trans-Anal Endoscopic Microsurgery (TEM)
LAMS	Lumen-Apposing Metal Stent
TTS	Through the Scope
OTSC	Over-the-Scope Clip
